# Monocular Near-Infrared Optical Tracking with Retroreflective Fiducial Markers for High-Accuracy Image-Guided Surgery

**DOI:** 10.3390/s26020357

**Published:** 2026-01-06

**Authors:** Javier Hernán Moviglia, Jan Stallkamp

**Affiliations:** Mannheim Institute for Intelligent Systems in Medicine, Medical Faculty Mannheim, Heidelberg University, Theodor-Kutzer-Ufer 1–3, 68167 Mannheim, Germany; jan.stallkamp@medma.uni-heidelberg.de

**Keywords:** optical tracking, surgical navigation, fiducial markers, image-guided surgery, high-accuracy tracking, retroreflective markers

## Abstract

**Highlights:**

**What are the main findings?**

- A monocular near-infrared optical tracking system using compact dodecahedral retroreflective markers achieves submillimeter and sub-degree accuracy.
- The ArUco MIP_36h12 fiducial family offers the best performance, with translational errors of 0.44 ± 0.20 mm and rotational errors of 0.35 ± 0.16° at distances of 30–70 cm, as measured from static position estimates.

**What are the implications of the main findings?**

- The system offers a compact, low-latency, and high-precision alternative to traditional multi-camera tracking setups in surgical navigation.
- Its sterilizable, CT/MRI-compatible marker design makes it suitable for integration into image-guided surgical workflows.

**Abstract:**

Image-guided surgical procedures demand tracking systems that combine high accuracy, low latency, and minimal footprint to ensure safe and precise navigation in the operating room. To address these requirements, we developed a monocular optical tracking system based on a single near-infrared camera with directional illumination and compact retroreflective markers designed for short-range measurement. Small dodecahedral markers carrying fiducial patterns on each face were fabricated to enable robust detection in confined and variably illuminated surgical environments. Their non-metallic construction ensures compatibility with CT and MRI, and they can be sterilized using standard autoclave procedures. Multiple fiducial families, detection strategies, and optical hardware configurations were systematically assessed to optimize accuracy, depth of field, and latency. Among the evaluated options, the ArUco MIP_36h12 family provided the best overall performance, yielding a translational error of 0.44 ± 0.20 mm and a rotational error of 0.35 ± 0.16° across a working distance of 30–70 cm, based on static position estimates, with a total system latency of 32 ± 8 ms. These results indicate that the proposed system offers a compact, versatile, and precise solution suitable for high-accuracy navigated and image-guided surgery.

## 1. Introduction

**Figure 1:**
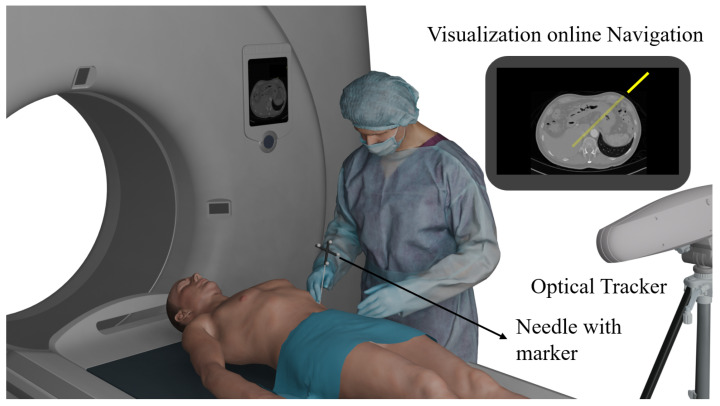
Example of a CT-guided needle biopsy procedure assisted by a stereoscopic IR optical tracking system, enabling real-time registration and visualization of the needle within the tomographic images.

Image-guided surgical navigation has become a cornerstone of modern interventions that utilize imaging modalities such as Computed Tomography (CT) and Magnetic Resonance Imaging (MRI). Central to these procedures is a high-performance tracking system that continuously monitors the position and orientation of surgical instruments. Such tracking enables accurate patient registration as described in [1,2]—the alignment of patient anatomy, surgical tools, and preoperative images—ensuring that navigation is precise and reliable. Fiducial marker-based registration, where physical or virtual reference points are attached to patients, instruments, or other objects to provide stable landmarks for the tracking system, remains widely adopted for its versatility, including use in soft tissue and ensuring reliable spatial references in navigation workflows, as illustrated by practical clinical cases in [3,4]. By integrating real-time tracking with fiducial-based registration, the system enables dynamic, accurate localization of instruments relative to anatomical targets, enhancing procedural safety, precision, and overall operational efficiency. In Figure 1, an example of a stereoscopic infrared (IR) optical tracking system implemented for image-guided navigation during a CT-guided needle biopsy procedure is shown. By employing a tracking system, it becomes possible to register the needle with respect to both the tomographic images and the patient’s position, thereby enabling real-time visualization of the needle within the image space.

Tracking systems in biomedical applications can be broadly classified into three categories: Optical Tracking Systems (OTSs), Electromagnetic Tracking Systems (EMTSs), and other systems primarily explored in research contexts [5], including Inertial Measurement Unit (IMU) sensors, wave-based technologies such as Ultra-Wideband (UWB), 5th Generation mobile networks (5G), Radio-Frequency Identification (RFID), WiFi, Bluetooth, and Global Positioning System (GPS), as well as mechanical, fiber-optic, or hybrid systems. EMTSs are incompatible with MRI environments due to interference from active electromagnetic components. Similarly, many alternative approaches in the third category are rarely used in clinical practice, as they often contain active elements or materials that are not MRI-compatible and remain largely experimental. Consequently, OTSs are the prevailing choice for image-guided procedures, offering high precision, reliability, and versatility across different surgical contexts.

Within optical tracking systems (OTSs), the two main categories are vision-based (visible-light) systems and infrared (IR) systems [5]. Among these, IR tracking systems currently represent the clinical standard, employing passive retroreflective markers detected by stereoscopic cameras and triangulation algorithms [6], as illustrated in Figure 1. They achieve submillimetric accuracy, as demonstrated in commercial platforms such as Polaris Vega (NDI Inc., Waterloo, ON, Canada) [7], OptiTrack (NaturalPoint Inc., Corvallis, OR, USA) [8], and Navient (ClaroNav Inc., Toronto, ON, Canada) [9], and are robust against mechanical, thermal, and optical perturbations. However, their adoption is constrained by high cost, a large physical footprint, minimum working distance requirements, and marker dimensions that can compromise ergonomics—particularly in procedures involving small instruments, as shown in Figure 1, where the marker size is compared to the needle. Vision-based systems, in contrast, can be divided into marker-based and markerless approaches; while markerless methods, typically based on camera-driven deep learning as in the Benchmark for 6D Object Pose Estimation (BOP) [10], impose heavy computational demands and operational complexity, marker-based approaches remain the most reliable and widely adopted solution in clinical practice, providing high accuracy, low latency, and the additional capability of supporting patient registration by aligning surgical instruments with preoperative imaging data. Marker-based systems commonly employ fiducial markers with square geometries, such as ArUco [11] and AprilTag [12], detected through corner identification algorithms followed by a Perspective-n-Point (PnP) computation, which estimates the three-dimensional pose of the marker relative to the camera using both the detected 2D features and the intrinsic calibration parameters.

To improve detection robustness across multiple spatial orientations, previous implementations have incorporated polyhedral structures with markers on each face. For example, ref. [13] evaluated different polyhedral configurations and identified the dodecahedral shape as the best-performing object, while also reporting that the pentagonal geometry of the ArUco marker improves performance compared to the classical square shape; however, this implementation is not fully optimized, and the observed improvement is marginal with respect to the conventional square marker. Similarly, ref. [14] demonstrated submillimetric precision using this dodecahedral shape with square fiducial markers. This type of dodecahedral geometric configuration, using square fiducial markers, has also been previously investigated for interfacing mechanisms that maneuver virtual minimally invasive surgical instruments [15]. Despite these advances, significant challenges remain unresolved, including ensuring sterility, minimizing marker size, extending the effective workspace, improving ergonomics, and maintaining robustness under variable illumination.

This work presents a monocular-camera-based tracking system designed to address these challenges. The system detects and estimates the pose of a dodecahedral fiducial marker with retroreflective surfaces, illuminated with directional infrared light to enhance robustness under variable lighting conditions. Although the use of retroreflective surfaces with fiducial markers has been previously explored, for example, in [16], to improve robustness under challenging lighting conditions, to the best of the authors’ knowledge, their application for workspace optimization, size selection, and clinical usability has not yet been investigated. ArUco and AprilTag markers were systematically evaluated under multiple parameter configurations, and the influence of optical lens and camera settings on system performance was analyzed. Additionally, a sterility protocol was developed.

This approach not only demonstrates a practical solution for real-time image-guided surgical navigation but also establishes a scalable framework that combines high accuracy, ergonomic design, and multimodal compatibility, addressing limitations inherent to existing tracking technologies. The system’s integration into a clinical workflow underscores its potential to improve procedural efficiency, patient safety, and accessibility in diverse surgical environments.

## 2. Materials and Methods

### 2.1. Physical System

**Figure 2:**
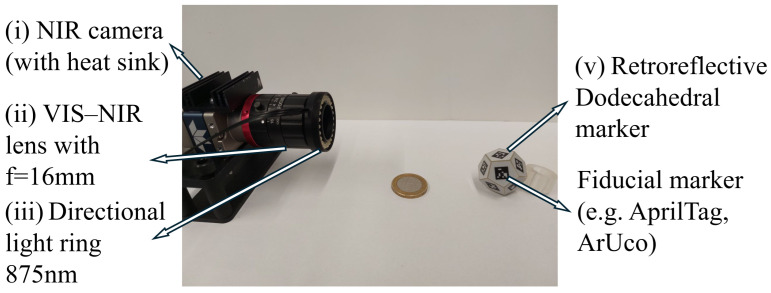
Hardware setup: NIR camera, 16 mm VIS–NIR lens, 875 nm light ring, and retroreflective dodecahedral marker.

The hardware components of the tracking system are shown in Figure 2. The system consists of the following: (i) a monocular monochromatic near-infrared (NIR) camera with a 2/3″ sensor (Teledyne DALSA Genie Nano M1930) with heat sink; (ii) a VIS–NIR lens with a focal length of 16 mm (TECHSPEC^®^ C-Series); (iii) a directional light ring centered at 875 nm (Vision Dimensions), mounted coaxially with the lens; and (iv) dodecahedral markers (12 faces) made of retroreflective material, each face carrying a square fiducial marker used for pose estimation. Image processing and marker detection are performed on a host computer equipped with an Intel vPro i7 CPU, enabling efficient execution of ArUco and AprilTag algorithms.

The retroreflective properties of the markers enhance the contrast between the background and the fiducials when illuminated by the coaxial light source. Since the illumination operates in the near-infrared (NIR) spectrum, it does not interfere with the user’s field of view (e.g., surgeon and patient) or with the ambient lighting in the operating room (OR).

#### 2.1.1. Optical System Setup

The appropriate selection of the camera and lens, along with their configuration parameters, is crucial for optimizing tracking range, latency, and accuracy. To determine the most suitable camera and lens, it was first necessary to define the desired tracking range together with the fiducial marker size.

On one hand, the minimum working distance is constrained by the safety distance between sterile and non-sterile components in an operating room. According to the Association of Surgical Technologists [17], a minimum distance of 300 mm must be maintained between the sterile field and non-sterile areas or objects to prevent contamination. In this context, the marker represents the sterile component, whereas the camera is non-sterile.

On the other hand, since the tracking system is intended for in situ use near the operator, assuming a camera position above the surgeon’s head and an ergonomic working height not exceeding the operator’s waist level, a maximum distance of at least 700 mm from the camera was considered. To balance compactness and reliable detection, a twelve-faced polyhedral marker with 10 mm edges was chosen to remain ergonomically small. In contrast, fiducial patterns of 7 mm were placed on each face to ensure sufficient feature size for accurate detection across the entire working range. This combination allows robust tracking in confined surgical environments without compromising operator comfort or detection precision.

**Figure 3:**
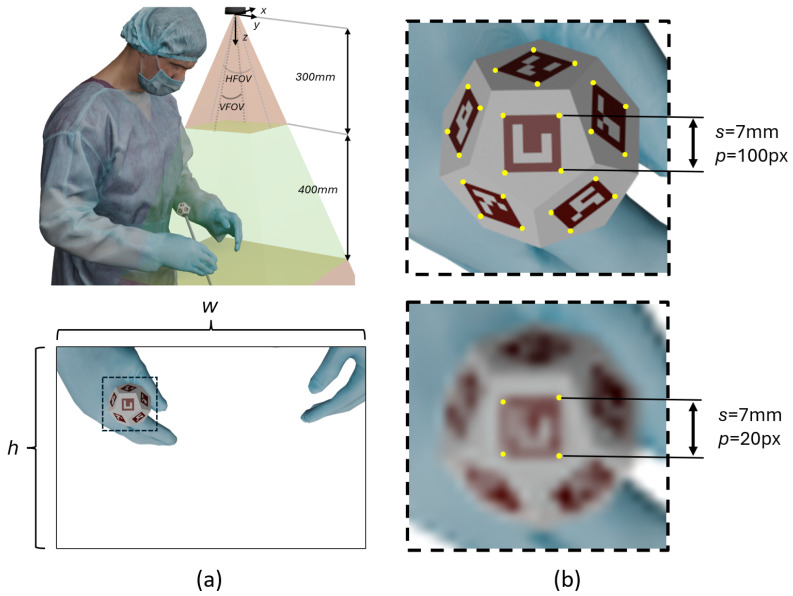
(**a**) Workspace of the tracking system, showing the optimal camera placement above the surgeon and the effective range for marker detection. (**b**) Enlarged view of the dodecahedral marker, illustrating the detection of marker corners for a high-resolution image (**top**) and a low-resolution image (**bottom**).

In Figure 3a, the workspace for which the tracking system was designed is shown along with the current camera field of view. Conversely, Figure 3b shows an enlarged image of the dodecahedral marker and illustrates the influence of the number of pixels representing each marker. In the lower-resolution image, the corners of the markers cannot be reliably detected. Since corner detection is a necessary step in the algorithm for estimating the dodecahedron’s position, undetected or inaccurately estimated corners lead to higher positional errors. This demonstrates a correlation between the number of pixels representing a marker and the achievable tracking accuracy.

Using Equation (1), which was derived to calculate the number of pixels *p* occupied longitudinally by a marker of side length *s* (in mm) at a given distance from the camera *z*, taking into account the camera resolution res and the horizontal and vertical fields of view (HFOV and VFOV, respectively), where the subscripts *w* and *h* correspond to the width and height dimensions, respectively, it was determined that the markers occupy 28 and 35 pixels, respectively, at their maximum effective ranges, while still achieving submillimetric accuracy, understood here as positional errors within the range of ±1 mm.

(1) $$\begin{array}{cc} p_{w} & =s_{w}\cdot \frac{res_{w}}{2z\;\tan\left(\frac{HFOV}{2}\right)}\\ p_{h} & =s_{h}\cdot \frac{res_{h}}{2z\;\tan\left(\frac{VFOV}{2}\right)} \end{array}$$

For the selected camera and lens, with a resolution of 1920 × 1200 pixels, a 2/3″ sensor (dimensions: 9.22 × 5.76 mm), and a focal length f=16 mm, the horizontal and vertical fields of view (HFOV and VFOV, respectively) were obtained using Equation (2), yielding HFOV = 32.1° and VFOV = 20.4°. At the maximum distance (700 mm), where the marker appears smallest in the image, the system achieves a side length of 33 pixels. This value falls within the previously calculated range, potentially ensuring submillimetric accuracy.

(2) $$\begin{array}{cc} HFOV & =2\;\arctan\left(\frac{sensor_{w}}{2f}\right)\\ VFOV & =2\;\arctan\left(\frac{sensor_{h}}{2f}\right) \end{array}$$

Achieving the required resolution at the maximum operating range does not guarantee submillimeter accuracy, since the captured image must remain both in focus and properly illuminated throughout the entire workspace. In previous work [18], we experimented with motorized zoom lenses and liquid lenses to adjust the focal length and/or focus based on the distance between the marker and the camera, thereby ensuring consistently sharp images. However, this approach requires recalibrating the camera for each focal length or focus configuration, which is both tedious and detrimental to system performance, as it reduces the effective tracking rate by necessitating multiple image acquisitions across the workspace.

To address these limitations, we used a single prime lens, which simplifies calibration to a single intrinsic matrix and leverages the superior optical quality of fixed-focal-length lenses. To keep the entire workspace in focus, the lens aperture is stopped down to increase the depth of field (DoF).

However, this introduces a fundamental trade-off: while a smaller aperture (higher f-number, *N*) reduces geometric blur from defocus, it increases diffraction blur. From diffraction theory, the radius *r* of the Airy disk—which defines the diffraction-limited spot size on the image sensor—is given by

(3) r≈1.22λN,

where λ is the wavelength of light, this radius determines the minimum achievable circle of confusion (CoC). A larger CoC smears the projected edges of the markers, increasing the uncertainty in sub-pixel edge detection and, consequently, the centroid localization error (see Equation (3)).

Therefore, selecting the f-number involves balancing two competing blur sources: geometric blur from insufficient DoF at wide apertures, and diffraction-induced blur at narrow apertures. Stopping down the aperture also reduces the light reaching the sensor. This loss can be compensated for by adjusting the gain and exposure time. To minimize image noise, the gain was fixed at 0 dB. Empirical evidence [14] indicates that an exposure time of 4 ms is sufficient for tracking dynamic objects; we therefore aimed for this value or lower. Note, however, that excessive illumination can saturate the retroreflective markers and distort their contours.

Considering all these factors—the theoretical trade-off between DoF and diffraction-limited sharpness, together with the practical constraints of illumination and noise—we evaluated several f-stop values (N=5.6,8,11,16). For each setting, the exposure time was adjusted to avoid saturation while maintaining adequate marker contrast. The configuration that delivered the highest practical centroid localization precision was selected, thereby optimizing the described balance.

Table 1 summarizes the key optical system specifications.

#### 2.1.2. Marker Design

The marker is a twelve-faced polyhedron with 10 mm edges, each face featuring a 7 mm fiducial marker. To determine the optimal material for printing these markers, various retroreflective technologies were evaluated.

Retroreflective Adhesive (RA) materials can be classified into three categories according to the European standard EN 12899-1 for fixed vertical traffic signs: RA1, RA2, and RA3, based on minimum retroreflectivity, with RA3 representing the highest performance. The standard also distinguishes between three criteria: Types A, B, and C, which refer to the manufacturing technology of the retroreflective material rather than its luminance level. These correspond, respectively, to the following: glass bead sheeting (Type A), high-performance microprismatic sheeting (Type B), and encapsulated high-intensity microprisms (Type C).

To identify the optimal material for the marker design, the fiducial markers were printed on different retroreflective materials, spanning various technologies and RA classes, as summarized in Table 2. Ultraviolet (UV) printing was used to ensure maximum adhesion and resolution on the retroreflective sheets.

**Figure 4:**
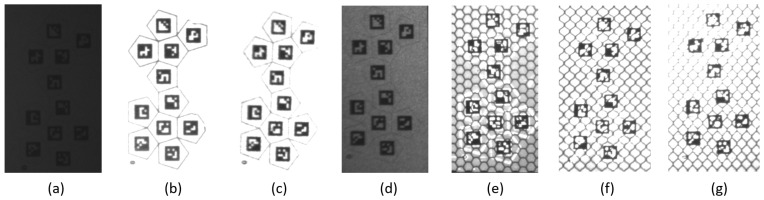
Fiducial markers printed on different retroreflective materials illuminated with IR light. Letters (**a**–**g**) correspond to the “Image” column in Table 2.

Table 2 lists all the materials tested, while Figure 4 shows the corresponding fiducial markers illuminated with IR light. Letters (a–g) in the figure correspond to the entries in the “Image” column of the table.

As shown in Figure 4, the best results were obtained with RA1 materials of Type A technology (images b and c), as the markers exhibit a homogeneous appearance and high-quality retroreflectivity. Since no significant differences were observed between the results of images b and c, the material Orafol ORALITE 5710 was randomly selected for further use. Fiducial markers were printed on this material and cut with a plotter to ensure precise positioning on the faces of the polyhedron, which was fabricated via SLA 3D printing with clear resin.

**Figure 5:**
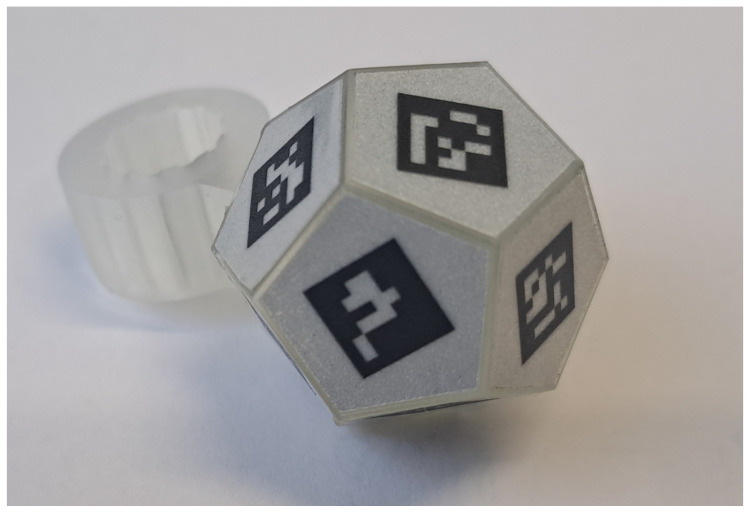
Final manufactured marker (here with ArUco fiducial markers).

Since the fabricated marker is not inherently sterile, it was rendered sterilizable by applying a thin coating of epoxy structural adhesive for medical technology (Loctite EA M-31 CL). This coating sealed the adhesive to the polyhedral surface while preserving the retroreflectivity of the fiducial marker print. The final manufactured marker is shown in Figure 5.

### 2.2. Algorithm

**Figure 6:**
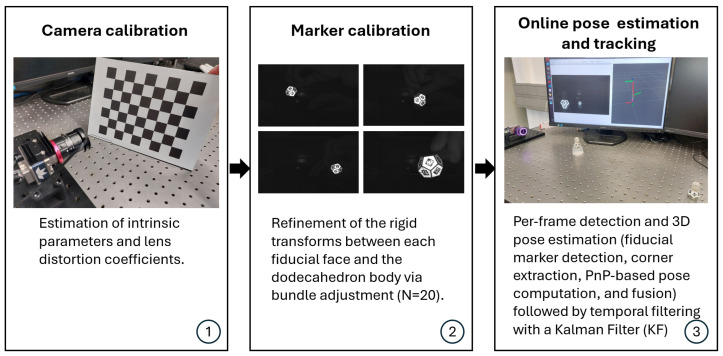
Processing pipeline for dodecahedral marker tracking: (1) camera calibration, (2) marker calibration, and (3) online pose estimation and tracking.

Once an image containing one or more dodecahedral markers is acquired, the system processes it to detect the square fiducial markers, extract their corner coordinates, and estimate their three-dimensional pose using the calibrated camera’s intrinsic parameters. The overall processing pipeline is summarized in Figure 6 and each stage is described in detail below:

#### 2.2.1. Camera Calibration

Accurate estimation of three-dimensional positions from two-dimensional image observations requires precise knowledge of the camera’s intrinsic and extrinsic parameters, as well as its lens distortion characteristics. Camera calibration was performed following the methodology proposed by Zhang [19], using multiple views of a chessboard (7 × 9, A4 size, checker size of 25 mm) to obtain the intrinsic matrix and distortion coefficients. During calibration, the parameters $k_{1}$, $k_{2}$, $p_{1}$, and $p_{2}$, as well as the constraint $f_{x}=f_{y}$ and fixed values for $c_{x}$ and $c_{y}$ (960 and 600, respectively, for a resolution of 1920 × 1200), corresponding to the OpenCV calibration model, were applied to minimize the reprojection error.

Homography refinement was applied to improve corner detection accuracy, particularly under partial visibility and occlusion. A notable feature of the calibration procedure is the fabrication of the chessboard according to the specifications described in Section 2.1.2, which ensures high contrast and optimal visibility, thereby improving the reliability and precision of the calibration results.

#### 2.2.2. Marker Calibration

Since each fiducial marker is manually attached to the faces of the dodecahedron, the relative placement of the markers with respect to the polyhedral body is subject to small errors. These misalignments introduce geometric inconsistencies that must be corrected to maximize the accuracy of 3D pose estimation. A bundle adjustment (BA) procedure is employed, which has been shown in the literature to achieve reliable results for multi-view pose refinement as demonstrated in [14,20].

The 2D observations of each marker are grouped across images, and outliers are removed based on initial reprojection errors. Using the remaining observations, initial camera poses for each timestamp are estimated via PnP. The BA optimization simultaneously refines:

- The 3D positions of the markers relative to the dodecahedron.
- The 6-DOF poses of the dodecahedron for each camera frame.

Let $p_{j}$ denote the pose of the *j*-th fiducial marker relative to the dodecahedron, and $p_{k}$ denote the pose of the dodecahedron relative to the camera in the *k*-th frame. For each observed corner $x_{i}$ of a marker, the reprojection error is the difference between its measured image location $O_{i}\left(x_{i}\right)$ and its predicted projection $I_{c}\left(u_{i}\left(p_{j};p_{k}\right)\right)$ through the camera intrinsics $I_{c}\left(\cdot \right)$. The overall BA objective is minimized as follows:

(4) $$E\left(\left\{p_{j},p_{k}\right\}\right)=\sum_{i}\sum_{j}\sum_{k}{∥I_{c}\left(u_{i}\left(p_{j};p_{k}\right)\right)-O_{i}\left(x_{i}\right)∥}^{2}\;$$

where the summations extend over all observed corners *i*, markers *j*, and camera frames *k*.

During optimization, one marker is typically fixed to eliminate global translational and rotational ambiguity. The remaining marker positions and camera poses are iteratively adjusted using a robust least-squares solver with a soft $L_{1}$ loss to reduce the influence of any residual outliers. Additionally, two constraints were introduced to prevent overfitting: the square shape of the markers, each with a side length of 7 mm, and their alignment with the twelve planes of the theoretical dodecahedron. The latter assumes accurate printing of the markers on the retroreflective material and precise 3D printing of the structure.

The outcome is a set of corrected marker positions that provide a consistent geometric model of the dodecahedron. These refined positions are subsequently used in online pose estimation to fuse observations from multiple faces into a single global pose of the polyhedron. The final optimization quality is evaluated via RMS reprojection errors, confirming that the refined model accurately reproduces the observed 2D marker projections.

#### 2.2.3. Per-Frame Detection and 3D Pose Estimation

Once the camera calibration matrix and its distortion coefficients, as well as the calibration of each fiducial marker on each face of the polyhedral marker, have been obtained, it is possible to proceed with the detection algorithm and the estimation of the 6D pose of the polyhedron (position and orientation) relative to the camera coordinate system. Two algorithms have been implemented for this purpose in their latest versions: ArUco (OpenCV 4.7.0) and AprilTag (version 3). These represent two of the fiducial marker types that have achieved the best performance in terms of latency, detection robustness, and accuracy [21].

Both algorithms detect the corners of the markers and the marker ID, although they use different detection approaches (ArUco [11]) and (AprilTag [12]). They also provide several configurable parameters to fine-tune detection (the complete list of parameters used for each algorithm is provided in Appendix A in Table A1 and Table A2, respectively). These parameters were tuned empirically, following the recommendations of the respective algorithm authors.

The algorithms were implemented within the Robot Operating System 2 (ROS2) framework, which allows runtime parameter modification via a graphical interface, visualization of detection and pose estimation results via its integrated tool RViz2, and scalability through intercommunication with other software or hardware components.

Once the marker corners are detected, the subset of markers corresponding to each board or dodecahedron is selected. At least two markers are required to resolve perspective ambiguities, since a single marker can yield two possible poses. The 3D pose of the board is then estimated using OpenCV’s solvePnP with the iterative Levenberg–Marquardt algorithm (SOLVEPNP_ITERATIVE). To improve accuracy, the initial pose is refined by minimizing the reprojection error of all detected marker corners. Only poses with sufficiently low reprojection error are retained for further processing and visualization.

To reduce jitter and ensure smooth tracking, the 6D pose estimations obtained from fiducial markers were filtered using a Kalman filter. It predicts the system’s expected state (pose) using a motion model and then corrects it with new measurements from the detection algorithm. For the position, a constant-velocity model with six state variables (position and velocity in *x*, *y*, and *z*) and three measurements (translation components) was used. For the orientation, an eight-state model was adopted to represent quaternion values and their dynamics, with four measurements corresponding to the quaternion components.

Process noise and measurement noise covariance matrices were tuned empirically, allowing a trade-off between responsiveness and smoothing. Additionally, quaternion normalization and sign disambiguation steps were implemented to ensure consistency across frames. The result is a continuous, stable trajectory of the detected marker board, which is then broadcast as a filtered transform in the ROS2 TF tree and published as a PoseStamped message for further use by other components of the system.

**Figure 7:**
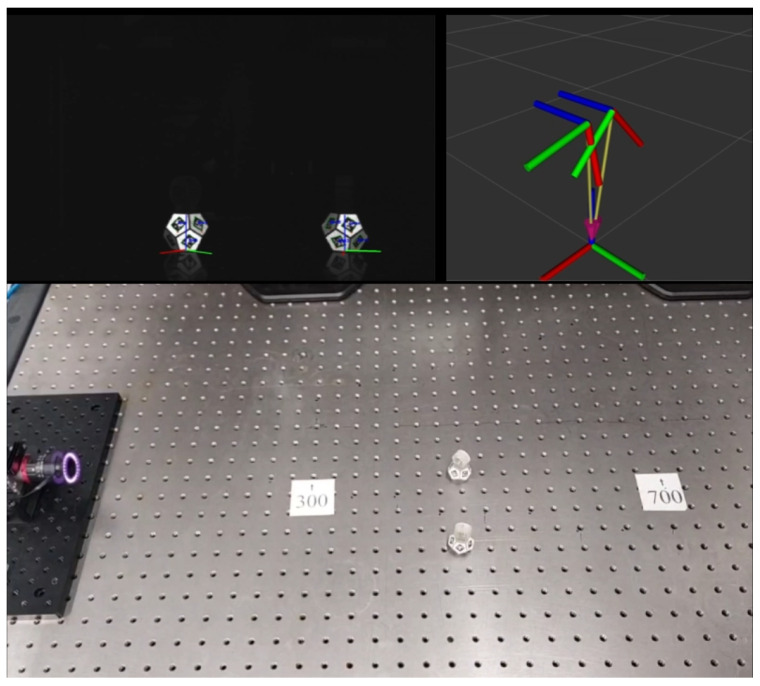
Tracking in operation and visualization of the 6D pose estimation in RViz from Video S1 (DemoTracking.mp4, Supplementary Materials).

Figure 7 shows a snapshot of the tracking in operation and the visualization of the 6D pose estimation in RViz.

## 3. Results

### 3.1. Evaluation of Accuracy and Precision in Static Positions

In Section 2.1.1, it was mentioned that the following f-stops were tested: 5.6, 8, 11, and 16. The corresponding exposure times were 0.5 ms, 1.5 ms, 1.6 ms, and 3 ms, respectively. The same camera calibration parameters were used for all cases, as variations in exposure time and/or aperture have negligible effects on them.

**Figure 8:**
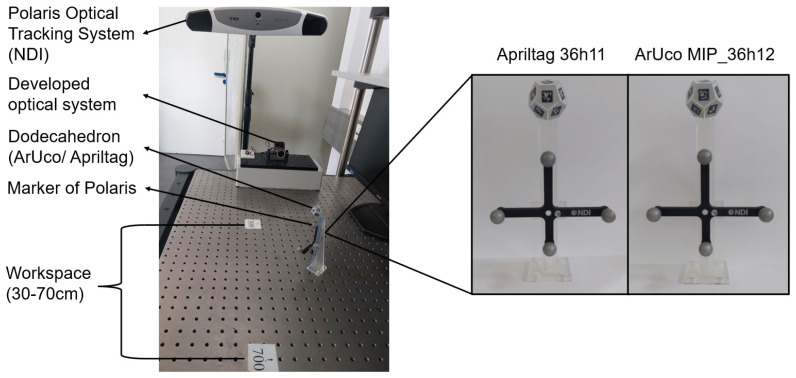
Experimental setup for accuracy measurement using the Polaris Optical Tracking System (NDI) in static positions (**left**). Dodecahedrons used for accuracy evaluation (**right**), each equipped with a Polaris marker composed of four retroreflective spheres. AprilTag 36h11 (**left**) and ArUco MIP_36h12 (**right**) are shown.

Two dodecahedra were designed, each incorporating fiducial markers from two different families: AprilTag 36h11 and ArUco MIP_36h12, as recommended by the authors of these fiducial systems. The dodecahedra were 3D-printed with a mount to hold a Polaris Vega VT Optical Tracking System (NDI) marker consisting of four retroreflective spheres, as shown in Figure 8. This marker was rigidly attached to the dodecahedron to assess the positional estimation accuracy of the developed optical tracking system throughout the workspace.

**Figure 9:**
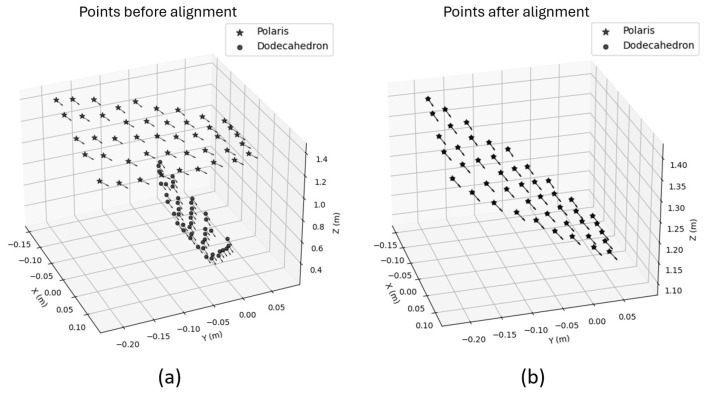
(**a**) Points before alignment at 45 different positions. Measurements from the Polaris tracking system (represented as “stars”) and from the developed tracking system (represented as “dots”) are shown. (**b**) Points after alignment using the Umeyama algorithm, RANSAC, and Wahba’s problem solution. In both panels, the Polaris measurements are indicated as “stars” and the developed system measurements as “dots”.

To perform this evaluation, 6D poses were measured using both the proposed optical system and the Polaris system (used as a reference) at five lateral positions and nine distances (30, 35, 40, 45, 50, 55, 60, 65, and 70 cm), resulting in a total of 45 uniformly distributed measurement points covering the entire workspace. At each point, the object was manually positioned and held stationary for the measurements. Once all poses were recorded, a robust alignment between the two coordinate systems was computed using a custom implementation based on the Umeyama algorithm with a fixed scale set to unity, combined with RANSAC to reject outliers. The resulting transformation was then refined using the inlier set. Orientation alignment was further improved by solving Wahba’s problem via singular value decomposition (SVD), minimizing the residual rotation between the systems (Figure 9).

**Figure 10:**
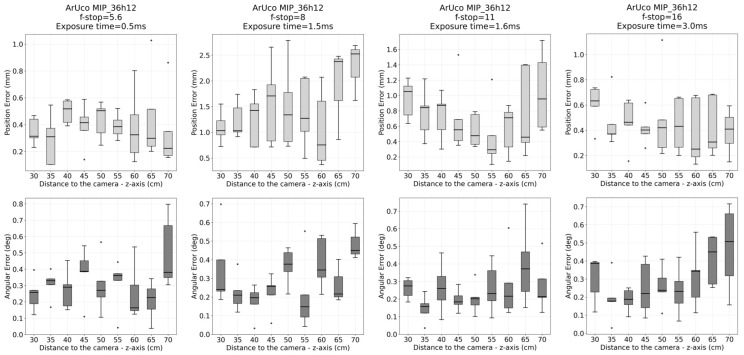
Aruco MIP_36h12: Translational and rotational error plots for all analyzed f-stop configurations (5.6, 8, 11, and 16). Results correspond to the comparison between the proposed optical tracking system and the Polaris reference system across the full workspace.

**Figure 11:**
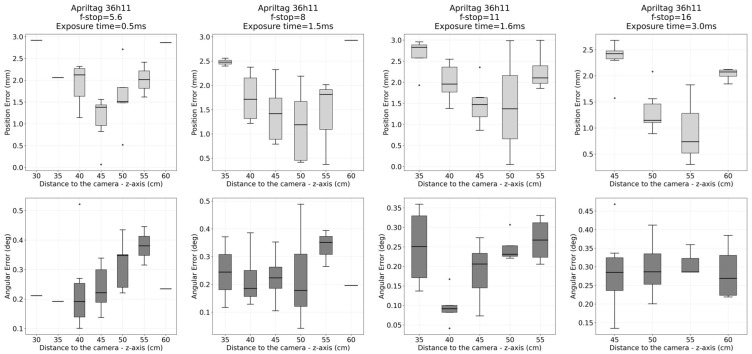
Apriltag 36h11: Translational and rotational error plots for all analyzed f-stop configurations (5.6, 8, 11, and 16). Results correspond to the comparison between the proposed optical tracking system and the Polaris reference system across the full workspace.

After alignment, the translational and angular errors were computed for each measurement point as the absolute deviations between the estimated and reference 6D poses. Figure 10 and Figure 11 show the translational and rotational error plots for all analyzed configurations, corresponding to the ArUco and AprilTag systems, respectively. Table 3 summarizes the accuracy results, which are represented by the RMS error. At the same time, precision is characterized by the standard deviation (Std. Dev.), both computed over the entire dataset for each configuration.

### 3.2. Evaluation of Accuracy and Precision in Dynamic Positions

**Figure 12:**
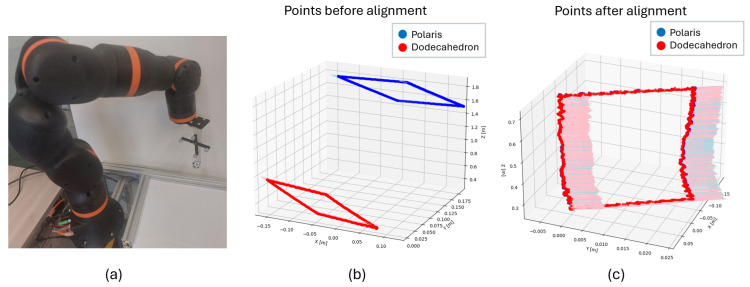
(**a**) Experimental setup for accuracy measurement in dynamic positions. (**b**) Points before alignment after performing the dynamic motion, measured with Polaris and the developed system. (**c**) Points after alignment using the Umeyama algorithm, ICP, and Wahba’s problem solution.

To evaluate accuracy and precision under dynamic conditions, a setup similar to that described for stationary positions was used, but with dynamic movements performed using an IGUS REBEL COBOT 6-DOF manipulator (Figure 12a). The defined trajectory was a rectangle of 40 cm × 15 cm in the camera’s xz-plane, covering the full depth of the tracking system workspace, with a constant speed of 50 mm/s. Ground truth measurements were also acquired using the Polaris Optical Tracking System.

Since submillimeter accuracy was already achieved in the previous analysis with markers ArUco MIP_36h12 at f/5.6 and f/16, the f/5.6 configuration was chosen here because it requires a shorter exposure time, making it more robust to motion blur. To evaluate accuracy and precision, an initial rough transformation based on timestamps was computed using the Umeyama algorithm, followed by the Iterative Closest Point (ICP) algorithm to refine the transformation. This two-step process compensates for the differing latencies of the two systems (the proposed optical system and Polaris), as identical timestamps do not necessarily correspond to measurements taken at the same time.

Once the transformation matrix was obtained using ICP, orientation alignment was further refined by solving Wahba’s problem via singular value decomposition (SVD), as performed in the previous stationary evaluation. Figure 12b,c show the results, including both positional data and quaternion-based orientations. The dynamic assessment yielded a translational RMS error of 0.55 mm with a standard deviation of 0.18 mm, and a rotational RMS error of 0.84° with a standard deviation of 0.45°.

### 3.3. System Stability over Extended Operation Time

To evaluate the system’s stability during extended operation and its susceptibility to thermal fluctuations in the camera and LEDs, two markers were placed approximately 50 cm from the camera’s *z*-axis, at the horizontal midline near the left and right edges of the camera’s field of view. Their positional and rotational values were recorded every 10 s for 120 min. All measurements were conducted at a room temperature of 20 °C.

**Figure 13:**
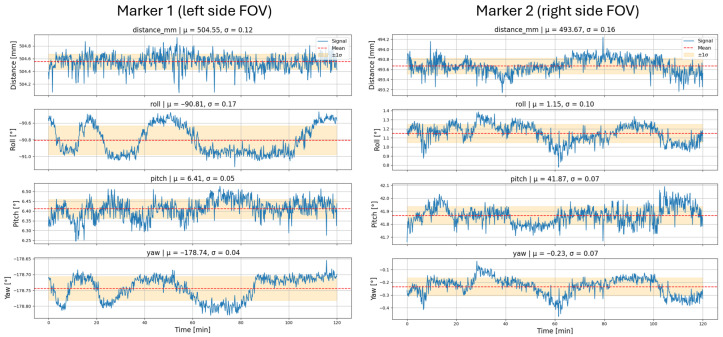
Variation of the Euclidean distance and orientation (roll, pitch, and yaw) of two markers fixed approximately 50 cm from the camera, positioned at the left and right edges of its field of view.

Figure 13 shows the evolution of the Euclidean distance from the camera to each marker, as well as the roll, pitch, and yaw angles over the 120 min. For Marker 1, located on the left side of the field of view, the standard deviations of the Euclidean distance, roll, pitch, and yaw were 0.12 mm, 0.17°, 0.05°, and 0.04°, respectively. For Marker 2, positioned on the right side of the field of view, the corresponding values were 0.16 mm, 0.10°, 0.07°, and 0.07°, respectively.

### 3.4. Latency

The total latency in an optical tracking system using fiducial markers (e.g., ArUco and Apriltag) represents the cumulative delay from image capture to pose availability. This end-to-end latency, denoted as $L_{\mathrm{total}}$, is the sum of the delays introduced by each stage of the processing pipeline. It begins with the camera’s exposure and readout time ($L_{\mathrm{cam}}$), followed by the transfer of the captured frame to system memory ($L_{\mathrm{trans}}$). The core computational stage for marker detection and pose estimation contributes to the processing latency ($L_{\mathrm{proc}}$), after which the application itself introduces a final delay ($L_{\mathrm{app}}$) to utilize the data.

The total system latency is thus modeled as follows:

(5) $$L_{\mathrm{total}}=L_{\mathrm{cam}}+L_{\mathrm{trans}}+L_{\mathrm{proc}}+L_{\mathrm{app}}$$

where

- $L_{\mathrm{cam}}$: Image capture latency.
- $L_{\mathrm{trans}}$: Data transfer latency.
- $L_{\mathrm{proc}}$: Computational latency for detection and pose estimation.
- $L_{\mathrm{app}}$: Application latency.

In this work, both the total latency and the processing latency were measured, as the latter is also commonly used as a performance metric in other optical tracking systems, as demonstrated in [13,14]. The total latency was measured using an IRLB8721 MOSFET to electronically trigger the illumination ring of the developed optical system and start a timer based on the PC’s monotonic steady clock. The timing was managed via USB Raw HID communication with a Teensy 4.0 microcontroller to minimize additional latency introduced by the controller itself. As soon as the ROS2-based application returned a pose estimate, the timer was stopped.

In contrast, the processing latency was measured using an internal clock within the same capture and pose estimation routine. This measurement procedure was repeated 15 times at random intervals for each evaluated configuration. The results, including the mean values and corresponding standard deviations, are summarized in Table 4.

### 3.5. Sterility

**Figure 14:**
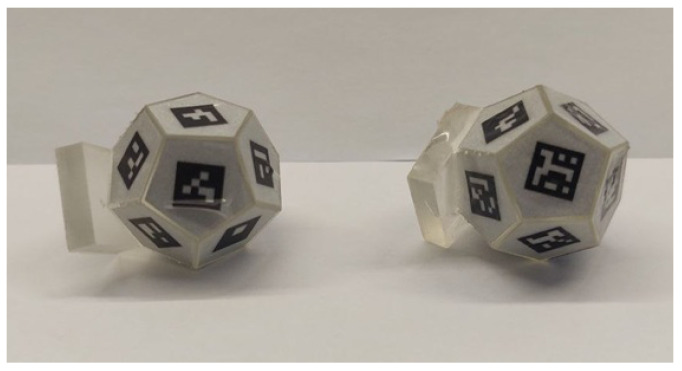
Dodecahedrons after autoclave sterilization. (**Left**): without protective coating; (**Right**): with protective coating. Note: the fiducial markers attached to both dodecahedrons are random.

The autoclave sterilization process was tested at 121°C and 1 bar for 15 min on a dodecahedron coated with the protective material Loctite EA M-31 CL, as well as on another without the protective layer. Figure 14 shows the condition of the dodecahedra after the autoclave process. The uncoated dodecahedron caused the retroreflective film to contract and deform, whereas the coated one exhibited no deformation and retained its retroreflective properties.

## 4. Discussion

Submillimeter accuracy (0.1–1 mm) and sub-degree angular precision (0.1–1°) were achieved across the workspace between 30 and 70 cm using ArUco markers, for both f/5.6 and f/16 apertures, with slightly lower positional error observed at f/5.6. In contrast, AprilTag markers did not achieve submillimeter accuracy and were not consistently detected across the evaluated range, although they exhibited a slight improvement in angular estimation. These differences can be attributed to the characteristics and configurability of each algorithm: the ArUco detector provides greater flexibility in detection parameters, allowing its performance to be tuned for different optical and illumination conditions, whereas AprilTag, with more limited configurability (see Appendix A, Table A1 and Table A2), prioritizes robustness to noise and contrast variations, which may constrain its performance at longer distances.

The failure to achieve submillimeter accuracy across all configurations with the ArUco marker at various apertures was primarily due to exposure time limitations. As noted in Section 2.1.1, excessive illumination of the retroreflective marker material can cause saturation and, consequently, contour distortion. Therefore, the exposure times for f/8 and f/11 were excessive, which explains the increase in positional error above 1 mm. Consequently, when designing the optical system following the proposed methodology, the shortest possible exposure time that still ensures reliable detection across the entire workspace should be considered, thereby maximizing accuracy for a given aperture.

Adopting ArUco at f/5.6 with an exposure time of 0.5 ms as the optimal configuration, and considering its standard deviation for both translational and angular errors, the translational error was measured as 0.44 ± 0.20 mm and the angular error as 0.35 ± 0.16°, rounded to two significant figures, based on static position estimates. It is worth noting that the Polaris Vega VT used for error measurements has an RMS volumetric error of 0.12–0.15 mm [22], which contributes marginally to the reported error. Importantly, the designed markers are significantly smaller than those of the Polaris Vega system, as illustrated in Figure 8. At the same time, the minimum tracking distance was reduced to 30 cm compared to 95 cm for the Polaris Vega [22]. As stated in [23], in instrument navigation, errors are typically on the order of a few millimeters, and submillimeter tracking accuracy is required to ensure that tracking errors do not dominate the overall system accuracy.

Furthermore, the evaluation of accuracy under dynamic conditions showed that the trajectories obtained with the proposed system closely matched those of the reference system, yielding a translational RMS error of 0.55 mm and a rotational RMS error of 0.84°. These results indicate that motion blur and rolling-shutter artifacts do not compromise submillimetric accuracy. In future work, acquisition times between the two systems will be synchronized using an external trigger to enable a more precise dynamic evaluation. Based on the results presented above, the designed system meets the accuracy requirements; however, further validation in clinical scenarios is planned to confirm its performance under real operating conditions.

During extended operation, the system demonstrated stability with variations considerably smaller than the accuracy measured under static conditions. Thermal fluctuations caused by components such as the camera and LEDs during prolonged use had a negligible effect on system stability. This was confirmed in tests conducted over 120 min, indicating that the system maintains reliable performance over extended durations. These results highlight the potential applicability of the proposed system in long-duration medical procedures.

The total latency using ArUco markers was approximately 32 ± 8 ms, while that using AprilTag markers was approximately 24 ± 4 ms. Latency depends strongly on detection parameter settings and hardware performance. Both algorithms run on CPU, so increased processing power or fine-tuning of detection parameters could reduce latency. Following the findings of [24] on latency in surgical AR environments, the 32 ms latency is well below the 100 ms threshold identified as critical for fine-motor task performance, ensuring effective real-time visual feedback. Moreover, this latency approaches the 17 ms reported for the Polaris Vega VT optical tracking system [22], a clinical reference standard.

Finally, the designed markers were validated for autoclave sterilization, rendering the system versatile for clinical use.

Overall, the proposed optical tracking system presents several advantages, including reduced marker size enabling single-camera operation, which decreases the operating room footprint and improves ergonomics; submillimeter accuracy with robustness to varying illumination conditions; low latency suitable for real-time guidance; reduced minimum working distance enabling on-site use; compatibility with autoclave sterilization; and safe use with imaging modalities such as CT and MRI due to the absence of metallic or ferromagnetic materials in the markers. Collectively, these characteristics indicate that the proposed system constitutes a viable alternative to conventional optical tracking solutions used in image-guided surgery.

The main limitations include the requirement for a clear line of sight, as is common in video-based optical tracking systems, and the need to change the lens to modify the tracking range. The former may be mitigated through multi-camera configurations or sensor-fusion strategies, depending on the specific clinical application’s requirements. The latter could be addressed by employing motorized optics with predefined focal and focus settings optimized for different workspace ranges. Future work will focus on adapting and implementing the proposed system in diverse clinical scenarios to assess its performance under real-world conditions further.

## 5. Conclusions

A highly accurate optical tracking system was developed using a monocular near-infrared camera and fiducial markers mounted on each face of a twelve-faced polyhedron with retroreflective film, specifically designed to meet the stringent requirements of image-guided surgical applications. The polyhedron is constructed entirely from non-metallic materials, making it compatible with multiple imaging modalities, including CT and MRI. Various hardware configurations and design parameters were systematically evaluated. Using MIP_36h12 ArUco markers on a dodecahedron with 10 mm edge length, a 16 mm lens at f/5.6, and an exposure time of 0.5 ms, the system achieved submillimeter translational accuracy of 0.44 ± 0.20 mm and sub-degree angular precision of 0.35 ± 0.16° over a 30–70 cm workspace, based on static position estimates, with a total latency of 32 ± 8 ms. Autoclave sterilization of the markers was validated, confirming compatibility with standard clinical protocols. The system’s compact form factor, extended tracking range, high accuracy, low latency, and versatility position it as a robust and clinically viable optical tracking solution for image-guided surgical procedures.

## Figures and Tables

**Table 1:** Camera, lens, and light specifications.

Component	Feature	Value
Camera	Type	NIR, Monochromatic
	Sensor size	9.22 × 5.76 mm
	Exposure Time	<4 ms
	Resolution	1920 × 1200
	FPS	80
	Analog Gain	0 dB
Lens	Focal Length	16 mm
	f-stop	5.6, 8, 11, 16
Light	Wavelength	875 nm
	Power	2 × 3 W
	Type	Directional Ring

**Table 2:** Retroreflective materials tested for fiducial marker printing.

Image	Material	Technology	Category (RA)	Type
(a)	White and black	Not retroreflective	-	-
(b)	Orafol ORALITE 5710	Glass Beads	RA1	A
(c)	3M™ Scotchlite™ 8906	Glass Beads	RA1	A
(d)	Orafol ORALITE 5600E	Glass Beads	RA1	A
(e)	Orafol ORALITE 5910	Microprismatic	RA2	B
(f)	Orafol ORALITE 6910	Full-cube microprismatic	RA3	C
(g)	3M™ Scotchlite™ 4090	Full-cube microprismatic	RA3	C

**Table 3:** Translational and angular RMS and standard deviation for ArUco and AprilTag under different imaging conditions.

	F-Stop = 5.6 Exp. Time = 0.5 ms		F-Stop = 8 Exp. Time = 1.5 ms	
	ArUco	AprilTag	ArUco	AprilTag
Translational RMS (mm)	0.438	1.872	1.559	1.772
Std. Dev. (mm)	0.195	0.712	0.667	0.744
Angular RMS (deg)	0.349	0.285	0.330	0.269
Std. Dev. (deg)	0.157	0.106	0.152	0.116
	**F-Stop = 11** **Exp. Time = 1.6 ms**		**F-Stop = 16** **Exp. Time = 3.0 ms**	
	**ArUco**	**AprilTag**	**ArUco**	**AprilTag**
Translational RMS (mm)	0.831	2.070	0.503	1.846
Std. Dev. (mm)	0.398	0.754	0.213	0.656
Angular RMS (deg)	0.290	0.221	0.327	0.304
Std. Dev. (deg)	0.140	0.088	0.156	0.078

**Table 4:** Measured total and processing latency for ArUco and AprilTag under different camera configurations.

Configuration	ArUco		AprilTag	
	Total Latency (ms)	Proc. Latency (ms)	Total Latency (ms)	Proc. Latency (ms)
F-stop = 5.6,				
Exp. Time = 0.5 ms	31.95 ± 8.31	10.08 ± 3.84	24.40 ± 3.17	4.76 ± 1.13
F-stop = 8,				
Exp. Time = 1.5 ms	30.28 ± 7.29	9.50 ± 3.27	22.75 ± 4.41	5.10 ± 1.32
F-stop = 11,				
Exp. Time = 1.6 ms	33.06 ± 7.87	9.90 ± 2.66	26.03 ± 3.08	4.55 ± 0.77
F-stop = 16,				
Exp. Time = 3 ms	30.83 ± 4.58	9.83 ± 1.83	24.07 ± 3.37	5.03 ± 1.13

## Data Availability

The original contributions presented in this study are included in the article. Further inquiries can be directed to the corresponding author.

## Supplementary Materials

The following supporting information can be downloaded at https://www.mdpi.com/article/10.3390/s26020357/s1, Video S1: DemoTracking.mp4.

## Abbreviations

The following abbreviations are used in this manuscript:

BA	Bundle Adjustment
BOP	Benchmark for 6D Object Pose Estimation
CoC	Circle of Confusion
CT	Computed Tomography
DoF	Depth of Field
EMTS	Electromagnetic Tracking Systems
GPS	Global Positioning System
HFOV	Horizontal Field of View
ICP	Iterative Closest Point
IMU	Inertial Measurement Unit
IR	Infrared
MRI	Magnetic Resonance Imaging
NIR	Near-Infrared
OR	Operating Room
OTS	Optical Tracking Systems
PnP	Perspective-n-Point
RA	Retroreflective Adhesive
RFID	Radio-Frequency Identification
RMS	Root Mean Square
ROS2	Robot Operating System 2
SVD	Singular Value Decomposition
UWB	Ultra-Wideband
UV	Ultraviolet
US	Ultrasound
VFOV	Vertical Field of View
VIS	Visible
5G	5th Generation mobile networks

## Appendix A

**Table A1:** Parameters used for ArUco marker detection. Listed values correspond to the settings used in the experiments reported in this work.

Parameter	Value
aruco_dictionary_id	DICT_ARUCO_MIP_36h12
adaptiveThreshWinSizeMin	3
adaptiveThreshWinSizeMax	60
adaptiveThreshWinSizeStep	10
adaptiveThreshConstant	7
minMarkerPerimeterRate	0.02
maxMarkerPerimeterRate	4.0
polygonalApproxAccuracyRate	0.03
minCornerDistanceRate	0.05
minDistanceToBorder	3
minMarkerDistanceRate	0.02
cornerRefinementMethod	CORNER_REFINE_CONTOUR
cornerRefinementWinSize	7
cornerRefinementMaxIterations	100
cornerRefinementMinAccuracy	$1\times 10^{-6}$
markerBorderBits	1
perspectiveRemovePixelPerCell	16
perspectiveRemoveIgnoredMarginPerCell	0.13
maxErroneousBitsInBorderRate	0.4
minOtsuStdDev	5.0
errorCorrectionRate	0.6

**Table A2:** Parameters used for AprilTag marker detection and pose estimation. Listed values correspond to the settings used in the experiments reported in this work.

Parameter	Value
tag_family	tag36h11
quad_decimate	1.5
quad_sigma	0.0
nthreads	4
refine_edges	true
decode_sharpening	0.1
debug	false
critical_rad	0.785
max_line_fit_mse	10.0
min_white_black_diff	40
